# Commentary: Hypnotic Medications and Suicide: Risk, Mechanisms, Mitigation, and the FDA

**DOI:** 10.3389/fpsyt.2016.00210

**Published:** 2017-01-13

**Authors:** Xiulu Ruan, Jin Jun Luo, Alan David Kaye

**Affiliations:** ^1^Department of Anesthesiology, Louisiana State University Health Science Center, New Orleans, LA, USA; ^2^Departments of Neurology and Pharmacology, Lewis Katz, School of Medicine at Temple University, Philadelphia, PA, USA

**Keywords:** suicide, hypnotics and sedatives, FDA, overdose, buprenorphine, opioid withdrawal syndrome

McCall and colleagues (1) recently published a review entitled “Hypnotic Medications and Suicide: Risk, Mechanisms, Mitigation, and the FDA” in the September 2016 issue of the American Journal of Psychiatry. They noted that although epidemiological studies have demonstrated that hypnotic sedatives are associated with an increased risk for suicide, none of these studies adequately have controlled for depression and/or other psychiatric disorders. However, McCall et al. conclude that their review findings indicate that hypnotic sedative medications are associated with suicidal ideation and that future studies should assess whether increases in suicidality result from CNS impairments from a given hypnotic medication or whether such medication decreases suicidality because of improvements in insomnia (1).

We believe there is a difference between the following two clinical situations, e.g., when suicide is induced by the recommended use of certain hypnotic medication for insomnia versus when the hypnotic sedative medication is used simply as a vehicle to commit suicide. Clearly, excessive use of any substance can potentially be harmful and even fatal. Pure water, for example, if taken in excessive quantity, may cause serious complications, including death (2). The first case of self-induced water intoxication was reported in 1938 by Barahal (3). Epidemiologically, several lines of accumulating evidence have indicated that polydipsia was found in 6–17% of chronic psychiatric patients. A total of 25–50% of psychiatric patients with polydipsia have been reported to have symptoms of water intoxication (4). Under such circumstances, therefore, the relationship between deaths and water intoxication is of causality rather than association. Yet, there are no such warnings as, “Water use may cause deaths” or “drinking water is associated with deaths” on any water bottles. Overwhelmed Web information is already a problem for patients to look for evidence about the drug they are taking, which may adversely impact their expectations about their improvement.

We question the true benefit of mingling every possible association conceivable of any drug into its lengthy package insert. We know that physicians typically do not read the entire prescribing information provided in the FDA-approved package insert (FDA-PI) (5). Nevertheless, such warnings are generalized and copied into the PIs of other drugs of the same class. Such narratives may potentially be used as evidence in court in medico-legal processes, even when such association may not even be significant or even true. For example, in the FDA-approved PI of buprenorphine, there is a statement: “Because of the partial agonist properties of buprenorphine, sublingual tablets may precipitate opioid withdrawal signs and symptoms …” (6). The term “opioid withdrawal” is actually mentioned over 20 times in the buprenorphine PI. Yet, both the preclinical and clinical data reviewed by van Niel et al. (7) clearly demonstrate that buprenorphine can be combined with a full MOP agonist, without precipitating opioid withdrawal.

Finally, we are concerned about placing every possible association into the FDA-PI can even do potential harm to certain patients (e.g., those who choose to read the PI) *via* the triggering of a nocebo effect, which occurs when the expectation of a negative outcome precipitates the corresponding symptom or leads to its exacerbation. Nocebo responses demonstrate the powerful interaction between the therapeutic context and the patient’s mind–brain interaction. Studies have shown that difference in disclosure information about adverse events results in different clinical responses (8). Rigorous previous research has suggested that providing patients with a detailed enumeration of every possible adverse event can actually increase such side effects (9). The dilemma is when the harmfulness of the nocebo effect may outweigh the good in proper disclosure of medical information to the patient, and where the duty to inform may therefore be suspended (10).

To minimize the harmful nocebo effect which might be brought about from the disclosure of all possible associations of hypnotics, such as that with suicide, we wonder if it might be a good and pragmatic idea to disclose those strong associations or clear causality, but withhold those trivial or weak associations which might potentially turn out to be more harmful in triggering a nocebo effect when disclosed in the FDA-PI.

## Author Contributions

XR: initial draft and final revision. JL: second revision, proofreading, and final revision. AK: third revision, proofreading, and final revision.

## Conflict of Interest Statement

The authors declare that the research was conducted in the absence of any commercial or financial relationships that could be construed as a potential conflict of interest.
